# Synthesis of novel carbon-supported iron oxide sorbents for adsorption of dye from aqueous solutions: equilibrium and flow-through studies

**DOI:** 10.1038/s41598-022-24257-8

**Published:** 2022-11-21

**Authors:** Siphesihle Praise-God Khumalo, David Lokhat, Kimbelin Chetty, Latisha Chetty

**Affiliations:** School of Chemical Engineering, Howard College Campus, University of KwaZulu-Natal, Durban, South Africa

**Keywords:** Chemical engineering, Chemical engineering

## Abstract

Textile effluents contain dyes that negatively affect water bodies and inhibit photosynthesis by reducing sunlight penetration. This study investigated the adsorption capacity of an iron oxide sorbent immobilised on naturally derived carbon foam for the removal of organic methylene blue dye from water. In this study, the carbon precursor and iron oxide precursor were mixed and carbonised in a single vessel. Baking and carbonization of the natural grain combination produce a porous structure that can act as an effective support for the iron oxide particles. The carbon foam prepared had a self-assembled structure with flour as a basic element. Sorbents of 6 weight (wt)%, 15 wt% iron, and a 0 wt% iron control sample were prepared. Transmission electron microscopy (TEM) and Brunauer–Emmett–Teller (BET) techniques were used to examine the synthesised carbon foam physical properties and surface morphology. The adsorption capabilities were investigated in batch tests by determining the effects of an increase in iron content, sorbent dosage, contact time, and dye concentration. Breakthrough curves were obtained by varying the height of the sorbent bed and varying the flowrate of the dye solution. A higher bed height corresponds to a greater amount of adsorbent. The breakthrough and equilibrium adsorption capacities were found to increase with increasing bed height. When the flow rate is high, the dye solution leaves the column before equilibrium, resulting in shorter breakthrough and saturation times. Higher bed heights and lower flow rates resulted in optimal dye removal in the flow through the system. Breakthrough time increases with increasing iron content. The 15 wt% iron sample displayed superior adsorption capabilities than the 6 wt% sample, while the 0 wt% iron control sample displayed minimal adsorptive capabilities. The pseudo-first order kinetic model was the best fit model for this study (R^2^ > 0.96), and the adsorption equilibrium is best described by the Freundlich isotherm (R^2^ > 0.99). The results showed that an iron oxide sorbent immobilised on carbon foam made from natural sources is a good adsorbent for removing methylene dye.

## Introduction

Water pollution is a global issue and one of the major contributors is the textile industry. The textile dyeing industry uses a lot of water during the different stages of dyeing and finishing, among other things^1^. Dyes’ nonbiodegradability and resistance to light and oxidising agents complicate the selection of an appropriate method for their removal. Furthermore, toxicity bioassays have shown that most dyes are toxic^2^. Because of reduced light penetration, dyes can have a significant impact on photosynthetic processes in aquatic life^2^. As a result, removing colour from waste effluents has become increasingly important for the environment^3^. Most industries use water bodies such as rivers and lakes for the disposal of their waste^4^. This waste is saturated with colorants, salts, and other toxicants, which alter the clarity and pH of the water. Some communities rely on rivers and lakes as sources of drinking water. The discharge of these untreated effluents can pose health risks when ingested by humans and animals. Therefore, the discovery and implementation of new methods of wastewater treatment is a high priority. Some dyes generate toxic, carcinogenic, and mutagenic intermediates through hydrolysis, oxidation, and other processes^5^. Because of this, it is important to get rid of dyes from wastewater before putting it back into the natural environment^6^.

Furthermore, dyes can evade conventional wastewater treatment methods because they are designed to withstand physicochemical and biological degradation^7^. Adsorption is an enticing, simple, and effective method of removing pollutants from wastewater. Low-cost adsorbents are made from low-cost materials or even waste and appear to be economically appealing for practical application^8^. Several materials have been used as adsorbents, including agricultural wastes, natural compounds, and activated carbon^9^. The removal of dyes using conventional waste management methods can prove challenging as dyes comprise complex molecular structures, which makes them non-biodegradable^10^, resistant to aerobic digestion, and stable to oxidising agents and light. Possible methods of dye removal include coagulation, froth floatation, chemical oxidation, and adsorption^11^. Adsorption is the most flexible method because it can be used on a wider range of compounds^12^.

Many methods have been used to remove dyes from coloured wastewaters, including coagulation/flocculation, biological treatment, ozonation, photocatalysis, filtration, electrochemical, membrane processing, adsorption, and others^13^. Thus, among other techniques, the adsorption process has been shown to be an effective technique due to its efficiency, capacity, and large-scale applicability for dye removal, as well as the potential for adsorbent regeneration, recovery, and recycling^14^. A variety of adsorbents have been tested for their ability to remove dye from wastewater. To obtain a high-performance adsorbent, it is critical to choose more efficient and less expensive adsorbents with higher adsorption ability^14^. A suitable adsorbent material should have a large surface area as well as a small volume^15^. Other characteristics must include high mechanical strength, chemical and thermal stability, high porosity, and small pore diameter, which results in more exposed surface area and thus suitable surface chemistry, resulting in high adsorption capacity^16^. The properties of mechanical and thermal strength, high surface area, suitable ordered structure, magnetic properties, optical properties, and the ability to manipulate them for required properties have all piqued the interest of researchers^17^. Nanoparticles are small particles with diameters ranging from 1 to 100 nm ^18^. They are suitable materials for a variety of commercial and domestic applications due to their unique properties, including catalysis, medical, imaging, agricultural, and engineering applications^17^. Nanoparticles are one of the most studied materials of the twentieth century because they have unique and new properties and can be used in many ways^18^.

This study intends to determine the adsorption efficiency of an iron oxide sorbent immobilised on naturally derived carbon foam for the removal of organic dye from an aqueous solution^19^. The effects of varying the iron content of the sorbent, contact time, dye concentrations, and sorbent dosage were studied. Also, the determination of saturation and breakthrough points of the sorbent were investigated. A carbon foam was synthesized, and its dye adsorption ability was investigated. The isotherm and kinetics of dye adsorption were thoroughly investigated.

This carbon source was tested as a model compound to prove the concept, particularly because it was easy to obtain and manipulate. The intention is to eventually progress to a different type of natural grain, one that is not used as a food source and can be used to produce the same or similar quality of carbon foam (as a support for the iron). The synthesis of the iron oxide on the carbon foam used in this study proceeds via a one-pot process where the carbon precursor and iron oxide precursor are combined and then carbonised together. When the natural grain mixture is baked and carbonized, it forms a porous structure that the iron oxide particles can use to stick to.

## Materials and methods

### Materials and chemicals

The following ingredients were used: flour (300 g), yeast (5 g), water (400 mL), iron nitrate crystals (4 mg), deionised water.

Iron nitrate of analytical grade purity and methylene blue dye were purchased from Sigma-Aldrich. The argon gas used for the carbonisation process was purchased from Afrox.

### Sorbent preparation

The natural grain mixture made of flour, water, and yeast was mixed in a large beaker for 10 min using the overhead stirrer. The mixture was then transferred to the aluminium trays. The sample was placed in an oven, which was set at 35 °C for 60 min to promote activation of the yeast. Thereafter, the sample was transferred to a second oven, which was set at 180 °C to bake. The oven rack was lined with aluminium foil to contain spills as the mixture rose significantly during the baking process. The second oven temperature was changed to 80 °C after 40 min, and the sample was left to dry in these conditions for 18 h. Carbonisation of the dried sample took place in the *U*-tube furnace under argon flow. A rotameter regulated the flow of argon gas. The analytical balance was used to measure how much iron was in the sorbent by weighing the samples.

### Spectrophotometric analysis

The UV–Vis spectrophotometer was switched on and given 15 min to warm up. The sorbent was removed from the treated solution through vacuum filtration. The setup included a Buchner flask and funnel. Stainless steel mesh was placed in the Buchner funnel as a filter. Cuvettes were used to test the filtered solutions, and the digital absorbance readings were recorded.

### Calibration of the Spectrophotometer

Methylene blue powder was mixed with deionised water to produce aqueous solutions of the following concentrations: (5, 10, 15, 20, 30, 40) mg/L. The spectrophotometer was switched on and allowed to warm up for 10–15 min. The spectrophotometric analysis was performed at the wavelength of maximum adsorption, which was 662 nm for methylene blue dye^20^. A cuvette filled with distilled water was used as the reference solution. This cuvette was inserted into the spectrophotometer and the device was set to show an absorbance value of zero. The cuvette was then removed. A second cuvette was filled with the 5 mg/L solutions, placed in the spectrophotometer and the device was run. The digital reading obtained, which indicated the amount of light absorbed by the solution, was recorded. For each concentration of methylene blue solution, all the steps were done again. For each sample, a new cuvette was used so that the solution wouldn’t get diluted.

### Batch tests

Glass beakers (50 mL) were used to conduct the iron content, sorbent dosage, and contact time tests. The beakers were covered with parafilm to avoid contamination. For the contact time test, a magnetic stirrer was employed to agitate solutions. The tests were carried out for each of the 3 samples at concentrations of dye solutions of 10 mg/L and 20 mg/L, respectively.

When the concentration of the solution after adsorption is known, the absorbance can be calculated. Absorbance is a measurement of the milligrams of dye adsorbed per gramme of sorbent. The following equation was used to determine the absorbance capacity of the sorbent^21^.

1 $${q}_{t}=\frac{{(C}_{0}-{C}_{t})\times V}{m},$$

2 $${q}_{e}=\frac{{(C}_{0}-{C}_{e})\times V}{m},$$

where q_t_ (mg/g) is an adsorption density, q_e_ (mg/g) is an adsorption density at equilibrium, C_o_ (mg/L) is the initial concentration of aqueous solution, C_t_ (mg/L) is the concentration of aqueous solution at time t, C_e_ (mg/L) is an equilibrium concentration of aqueous solution, V (L) is the volume of solution, and m (g) is the mass of adsorbent.

To obtain the percentage removal of dye, the initial and final concentration of the solution were required. The calculation was carried out using the following equation^21^:

3 $$\%R=\frac{({C}_{0}-{C}_{t})\times 100}{{C}_{0}},$$

where % R is the percentage of Methylene blue dye removed, C_o_ (mg/L) is the initial concentration of aqueous solution, and C_t_ (mg/L) is the concentration of aqueous solution at time t.

### Flow-through Tests

**Figure 1:**
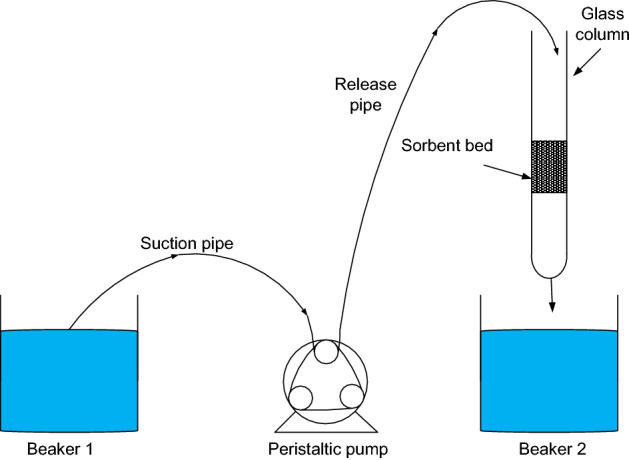
Flow-through apparatus setup.

A flow-through apparatus was used to determine the breakthrough and saturation points of the sorbent. Figure 1 below shows the setup of the apparatus. The suction pipe of the peristaltic pump was placed in a beaker filled with a 10 mg/L solution, and the release pipe was placed at the top of the glass column. Another beaker was placed at the exit point of the glass column to collect the treated water. This set-up made sure that the solution was pumped from beaker 1 to the glass column at a certain flow rate, where it went through the sorbent bed and ended up in beaker 2.

For the breakthrough tests, the effluent from the glass tube was tested at regular intervals to find out when the concentration of the effluent started to rise, or when the breakthrough happened, and when the sorbent reached its saturation point.

## Results and discussion

### Preparation of sorbent

Preparation of the sorbet was carried out according to the procedure outlined^22^. To determine the effect of iron content on the adsorption process, the samples were required to comprise different quantities of metal loadings. This was achieved by varying the amount of iron nitrate added to each sample. The iron nitrate was thermally decomposed to form iron oxide through a pyrolysis process under argon flow. A scaling calculation was used to obtain the final weight percentage of iron in the samples. Three samples were prepared. Sample A contained 6 wt. wt. Sample B contained fifteen wt% iron, and sample C was produced as a control sample, which contained no iron. The natural grain mixture served as a carbon source due to the glucose and protein it contains. Carbonisation under argon flow transformed the mixture into the desired sorbent. The following equation shows the reaction that occurred during the carbonisation process.

4 $$\left( {{\text{C}}_{{6}} {\text{H}}_{{{1}0}} {\text{O}}_{{5}} } \right){\text{n }}\left( {{\text{starch}}} \right) \, \to {\text{ 6n C}} + {\text{5n H}}_{{2}} {\text{O}}.$$

The samples exhibited visual differences after the baking process and before carbonisation. A colour difference was observed. The 0 wt% sample, which contained no iron, was the lightest in colour and the most brittle. After the process of carbonisation, the samples became visually identical, black in colour, and resembled charcoal. The carbon foam was very light and had a soft, powdery feel, which made it easy to break up into smaller pieces.

### Advantages and disadvantages

Nanomaterials offer excellent antifouling capabilities for many membranes and applications. Iron oxide gives iron immobilised in natural grain-based carbon foam both mechanical strength and stability. Since adsorption is a surface event, the nanoparticles’ presence increases the surface area^23^. If an abundant source of the natural grain precursor material can be identified, it has the potential to be a sustainable sorbent. Since it has less surface area than activated carbon, this could make it harder to sell.

### Characterisation of an adsorbent

Table 1 displays the findings of the investigations into the BET surface area, micropore volume, and pore radius. The results provide light on the pore structure of the carbon foam made from natural grains. The surface area of the samples with 15 wt% and 6 wt% iron is greater than that of the sample with 0 wt% iron. Additionally, the micropore volume of both 6 wt% and 15 wt% of iron was greater than 0 wt% of iron content of 0 wt%. The carbon foam has increased surface area, micropore volume. The presence of iron enhances the action of the yeast, i.e. the consumption of sugars in the grain mixture and the production of carbon dioxide, which is responsible for developing the pore structure in the carbonised material.

**Table 1:** BET analyses.

	0 wt% iron	6 wt% iron	15 wt% iron
BET surface area m^2^/g	0.0211	4.5464	3.7578
Average pore volume cm^3^/g	0.000014	0.001145	0.001211
Average pore diameter	25.8910	12.2719	11.985
Average hydraulic pore radius	0	3.7915	4.7918

**Figure 2:**
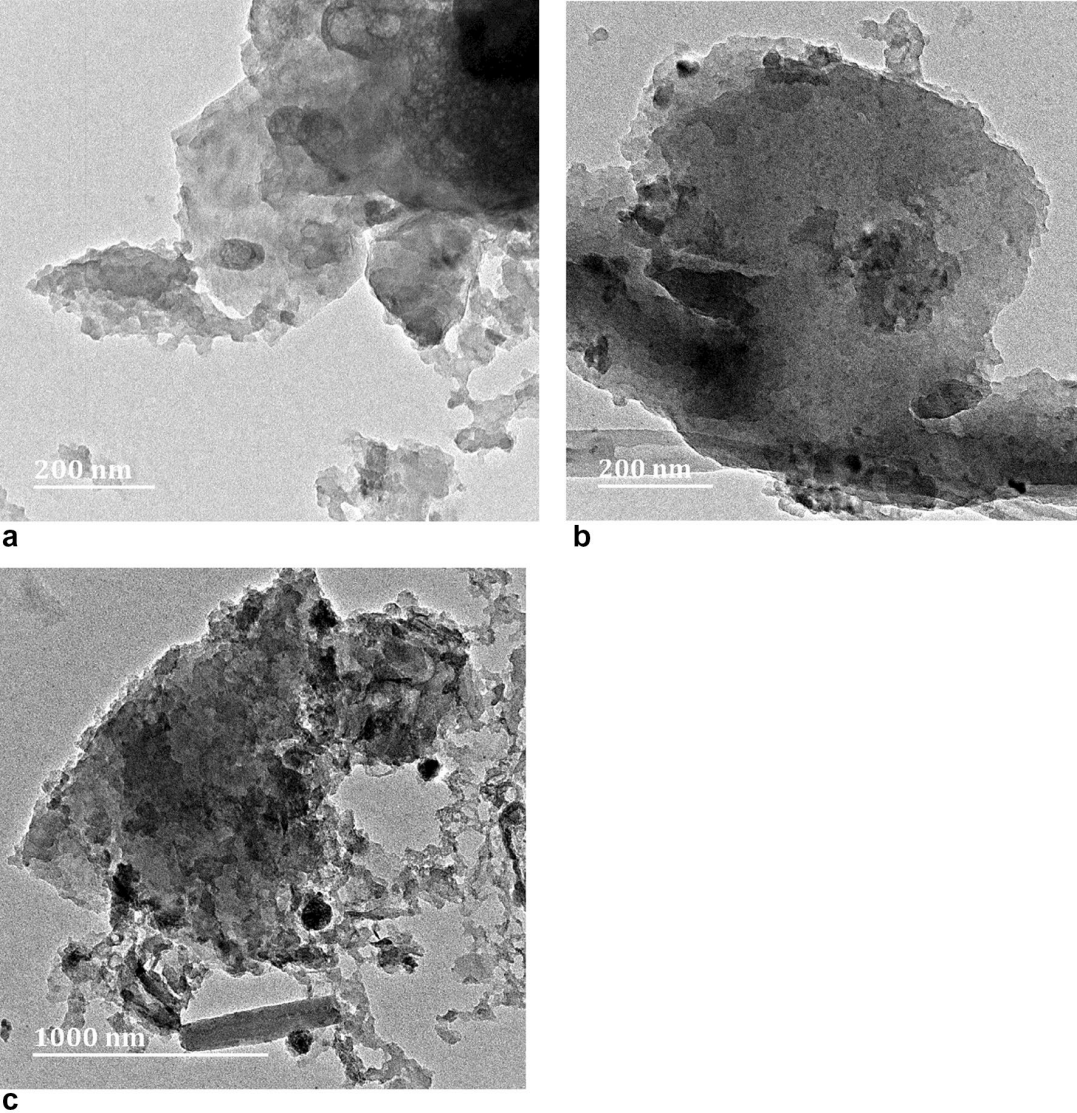
MET image conducted on a Jeol 2100 HRTEM operating at 200 kV (**a**) cabron foam with 0% wt iron (**b**) carbon foam with 6 wt% iron (**c**) carbon foam with 15 wt% iron.

The TEM micrographs of the natural grain carbon foam are shown in Fig. 2. It is evident that the addition of iron nitrate particles to the grain carbon foam resulted in a more amorphous and porous structure. The material surface possessed geometrical characteristics with an uneven shape, big agglomerates, and a rugged surface, which would provide additional sites for adsorbing heavy metal ions. These attributes were shown to be advantageous for the synthesised carbon foam.

### Effect of iron content

**Figure 3:**
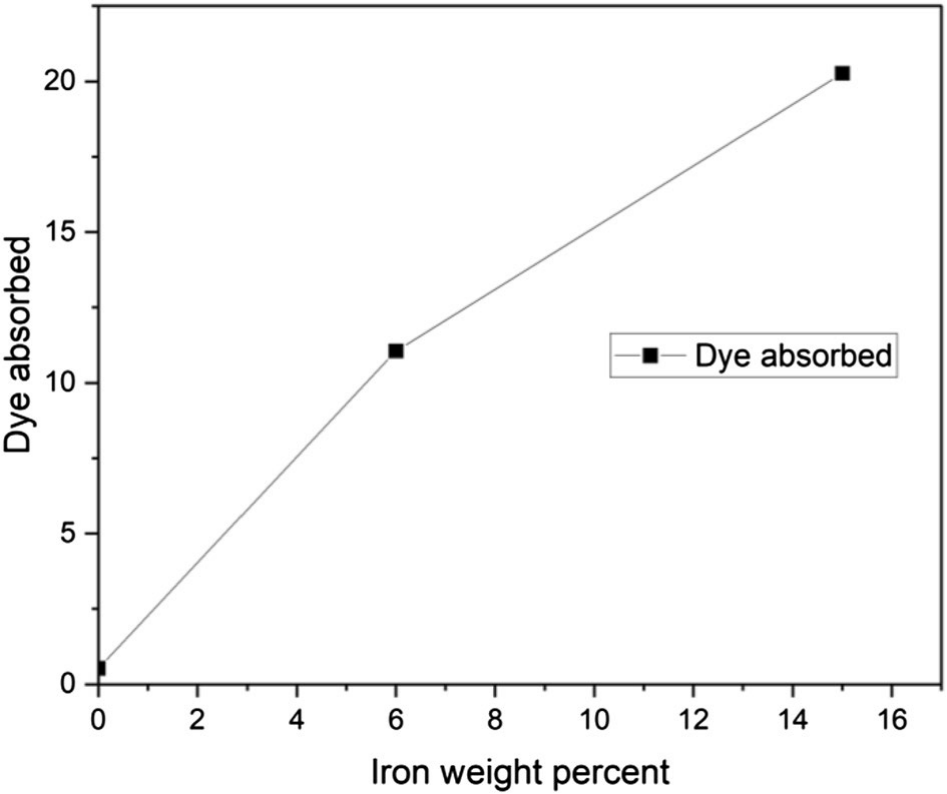
Effect of the iron content of the carbon foam at sorbent dosage of 20 mg, concentration of 10 mg/L, and contact time of 30 min.

Three samples of 0 wt%, 6 wt%, and 15 wt% iron on carbon foam of 20 mg were investigated using 10 mg/L and 20 mL of methylene blue dye. The contact time for each sample was 30 min. Thereafter, the solutions were vacuum filtered and absorbance readings were taken using the spectrophotometer. A control sample was used to determine the adsorption efficiency of the carbon support. Figure 3 shows that the control sample adsorbed less than 1% of the dye after 30 min, whereas the 6 wt% iron sample adsorbed 11.05% and the 15 wt% iron sample adsorbed 20.26%. This miniature effect on dye removal indicates that the carbon support was beginning to absorb after 30 min. During the same time period, the 15 wt 1% iron sample adsorbed approximately 9.21% more dye than the 6 wt% iron sample. This suggested that an increase in iron content within the sample increased the adsorptive properties of the sorbent^24^. However, studies carried out by Taromi and Kaliaguine in 2018 showed that if the metal content of the sample is excessive, then agglomeration may occur, which increases the size of the iron particles, thereby reducing the surface area^25^. Because of this, metal oxide is wasted, and the ability of a sorbent to take up water is also reduced.

**Figure 4:**
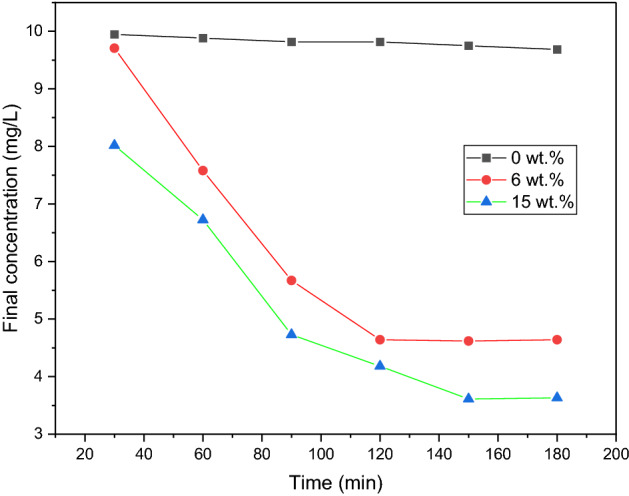
The leaching of iron ions, concentration of 10 mg/L, and contact time of 180 min.

Figure 4 displays the concentration of iron ions as a function of reaction time. The decrease in iron ion leaching as the reaction progressed suggests that iron ions in the solution may be redeposited on the iron-carbon foam surface^26^. At the end of Fig. 4, the variation of iron ion concentration with reaction time is depicted. The decrease in iron ion leaching as the reaction progressed suggests that iron ions in the solution may be redeposited on the iron-carbon foam surface. At the conclusion of the reaction (180 min), iron leaching was less than 3.61 mg/L for a 15 wt% sample and 4.64 mg/L for a 6 wt% sample, with a negligible difference from a 0 wt% iron control sample. This low level of leaching showed the stability and reusability of the iron-carbon foam particle electrodes, which was in line with the BET and TEM analyses.

### Effect of sorbent dosage

**Figure 5:**
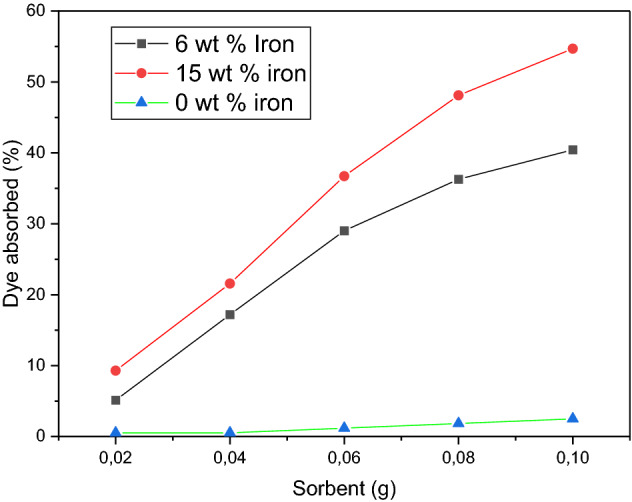
Effect of the iron content of the carbon foam at sorbent dosage, concentration of 10 mg/L, and contact time of 30 min.

Sorbent dosage test experimental design was conducted for 20, 40, 60, 80 and 100 mg of sorbent dosage. The concentrations of 10 mg/L and 20 mg/L methylene blue dye were utilised with a volume of 20 mL and contact time of 180 min. Three absorbance readings were taken for each filtered solution, and an average value was calculated. An increase in the amount of sorbent added to the aqueous solution improved the percentage of dye adsorbed over a period of 180 min for all the samples, see Fig. 5^27^. For the 6 wt% sample, at 20 mg of sorbent, 5.13% of the dye was adsorbed from the 10 mg/L solution. This percentage increased to 40.44% when a 100 mg dose was used. The rise in adsorption can be accredited to the increased availability of adsorptive sites that is the resultant of a higher dosage^28^. Initially, the removal of dye is rapid as the dosage increases from 20 to 80 mg; thereafter, there is an observable decrease in gradient. Such behaviour implies a decrease in adsorption efficiency. The adsorption efficiency of a sorbent is known to decrease when the sorbent approaches its saturation point. The driving force for adsorption declines as the dye molecules accumulate within the pores of the sorbent. This lessens the intensity of the attractive forces between the dye molecules and the sorbent, resulting in adsorption occurring at a slower pace. Figure 5 shows that samples of 6 wt% and 15 wt% displayed similar trends. The deviation between the samples was the amount of dye removed. A sample of 15 wt.% of iron, which consisted of a higher metal loading than the sample of 6 wt% of iron, adsorbed significantly more dye. The largest difference occurred at a sorbent dose of 100 mg, where 15 wt% of the iron adsorbed 54.69% of the dye. This was a deviation of approximately 14.25%. Therefore, the sample of 15 wt% of iron had a higher adsorptive capacity due to its higher metal loading. The control sample showed a slight increase in adsorption with increasing dosage. However, the highest dosage of 100 mg resulted in only 2.5% of the dye being removed. This indicated that the carbon support did not have a major impact on the adsorption efficiency of the sorbent. Adsorbent dosage increases dye adsorption due to increased adsorbent surface and the availability of more adsorption sites^29^. When the adsorption capacity was expressed in milligrams adsorbed per gramme of material, the capacity decreased with increasing adsorbent amount. It happens when adsorption sites overlap or group together, which makes less adsorbent surface area available to the dye and makes the length of the dye's path of diffusion longer^30^.

### Effect of contact time

**Figure 6:**
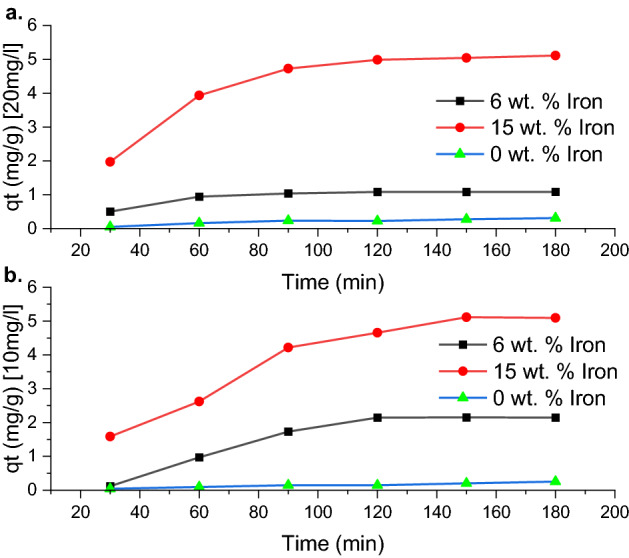
Effect of Iron content on carbon foam at sorbent of 50 mg, 10 mg/L dye concentration and contact time of 180 min and Effect of Iron content on carbon foam at sorbent of 20 mg, 100 mg/L dye concentration and contact time of 180 min.

Figure 6 displays the effect of contact time and concentration on the adsorption of methylene blue dye per gramme of sorbent. It was found that absorbance increased with increasing contact time. For the 10 mg/L solution, a steady increase in dye removal was observed during the first 90 min, after which the slope of the graph lessened and eventually levelled off at 120 min. Since the sorbent initially has no dye coating its surface, there are strong attractive forces between the surface of the sorbent and the dye molecules. As the sorbent becomes saturated, the large driving force for adsorption diminishes, and the adsorption efficiency decreases after 90 min. When the sorbent reached its saturation point at 120 min and an absorbance value of approximately 2.14 mg/g, no further adsorption took place. This is illustrated by the horizontal line displayed from the 120 min mark to the 180 min mark. The 20 mg/L solution shows higher absorbance during the first 90 min, see Fig. 6. This is a result of the increased number of dye molecules in the solution. The larger concentration gradient promoted more rapid adsorption. Before the sorbent became saturated, the adsorption efficiency for the 20 mg/L solution was much higher than the maximum efficiency for the 10 mg/L solution. This rise with increasing concentration is the result of an increased driving force for adsorption. This test showed that a solution with a higher concentration made the sorbent work better because it took less time for the sorbent to reach its maximum dye-removing ability.

**Figure 7:**
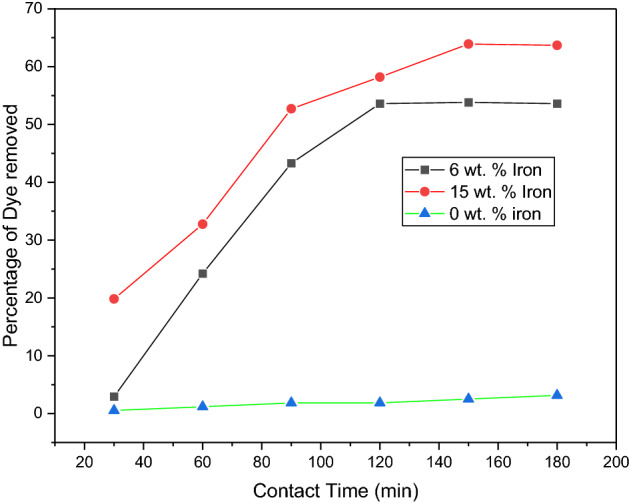
Effect of contact time, sorbent dosage (50 mg), concentration of dye solution (10 mg/L), and volume of solution (20 mL).

The percentage of dye adsorbed for each of the samples is shown in Fig. 7. A distinct difference in dye removal is noted for each of the 3 samples. As shown by the iron content test, the 15 wt% iron sample had the highest percentage removal of 63.68% after 180 min. Sample A removed 53.60% of the dye, and sample C continued to display minimal adsorption capabilities with a dye removal percentage of just 3.14 The sorbent dosage was kept constant for this test at 20 mg, and it was noted that the saturation point differed for samples with 6 wt% and 15 wt% of iron. Sample A reached saturation at 120 min, while Sample B reached its saturation point at 150 min. This showed that an increase in iron content not only increases the amount of dye removed per gramme of sorbent but also extends the lifespan of the sorbent.

### Adsorption kinetics

Adsorption kinetics were investigated to determine the relationship between contact time and dye uptake. At a constant temperature of 25 °C, the initial concentration influence of the reagent on the adsorption kinetics was investigated.

### Pseudo-first and pseudo-second order equations

For the investigation of modelling of adsorption kinetics, Lager Gren pseudo-first order and pseudo-second order models were used for the investigation. The nonlinear form of pseudo-first order is given by the following equation:

5 $${q}_{t}={q}_{e}\left(1-{e}^{-{K}_{1}t}\right).$$

**Figure 8:**
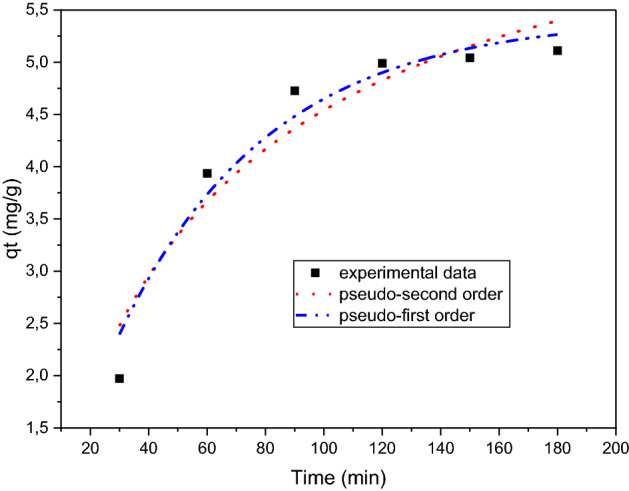
Nonlinear fit of pseudo-first order and pseudo-second order for dye adsorption on carbon foam with 15 wt% iron, 20 mg/L solution, initial concentration of 10 mg/L.

The nonlinear form of pseudo-second order is given by the following equation:

6 $${q}_{t}=\frac{{{q}_{e}}^{2}{K}_{2}t}{1+{K}_{2}{q}_{e}t},$$

where q_t_ (mg/g) is the amount absorbed at time t, q_e_ (mg/g) is the amount remaining after equilibrium of adsorption, and K_1_ and K_2 are_ the pseudo-first and pseudo-second order model rate constants, expressed in min^-1^ and g/mg/min, respectively. Tables 2,3 illustrate the calculated values of q_e,_ K_1,_ K_2_, and the regression coefficient R^2^ values. Figure 8 shows the plot of pseudo-first order and pseudo-send order. The validity of a model is judged by evaluating correlation coefficients R^2^ and the comparability of experimental and calculated values of q_e_. Therefore, a pseudo-first order model is the best fit for the adsorption process of methylene blue on carbon foam.

**Table 2:** Pseudo-first order parameters.

Pseudo first order				
Iron wt%	Exp. qe	Calc. qe	K_1_	R^2^
**10 mg/L solution concentration**				
6%	2.263	3.501	0.006	0.875
15%	5.390	6.252	0.011	0.967
**20 mg/L solution concentration**				
6%	1.107	1.129	0.245	0.943
15%	5.355	5.404	0.019	0.957
0%	0.391	0.423	0.007	0.937

**Table 3:** Pseudo second order parameters.

Pseudo second order				
Iron wt%	Exp. qe	Calc. qe	K_2_	R^2^
**10 mg/L solution concentration**				
6%	2.263	6.088	0.001	0.870
15%	5.239	9.274	0.001	0.958
0%		2.012	3.795	0.965
**20 mg/L solution concentration**				
6%	1.107	1.393	0.019	0.888
15%	5.355	7.062	0.003	0.922
0%	0.391	0.423	0.007	0.934

### Intraparticle diffusion

**Figure 9:**
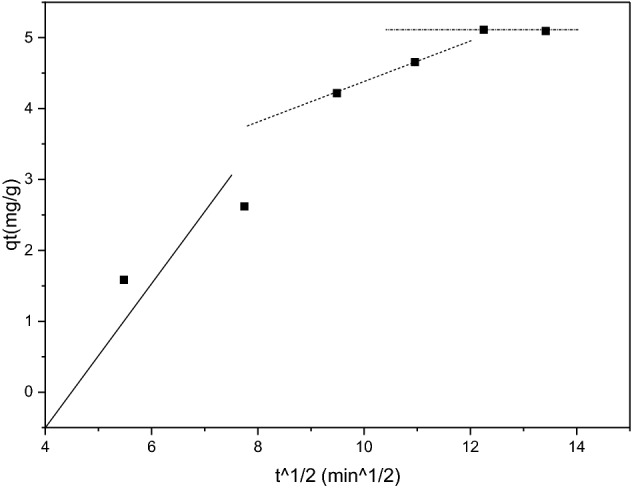
Plot of the intraparticle diffusion model for methylene blue onto iron supported by carbon foam (50 mg), 10 mg/L solution, room temperature.

The intraparticle diffusion resistance was evaluated using the intraparticle particle diffusion model by Weber and Morris^31^, given by the following equation:

7 $${q}_{t}={k}_{id}{t}^{1/2}+c,$$

where q_t_ (mg/g) is the adsorption amount at time t (min), k_id_ (mg/g/min^1/2^) is the adsorption rate constant of the intraparticle diffusion model, and c is a constant related to the thickness of the boundary layer. The plot of qt versus t^1/2^ is the sole rate-limiting step of the adsorption process if it is linear and passes through the origin^32^. When the lines of uptake pass through the origin, the rate-controlling step is intraparticle diffusion. When the plots do not pass through the origin, it indicates that the intraparticle diffusion is not the only rate-limiting step, but that other kinetic models may also control the rate of adsorption, all of which may be operating concurrently^10^. The plots of intraparticle diffusion illustrated in Fig. 9 are non-linear and do not pass through the origin. This indicates that intraparticle diffusion was not the only rate-limiting step of the adsorption process of methylene blue onto iron supported by carbon foam but that other mechanisms may also control the rate of the adsorption, all of which may be operating simultaneously^33^.

### Equilibrium adsorption

Adsorption equilibrium occurs between the adsorbed molecules and the adsorbent surface when an adsorbate is in contact with the adsorbent. The adsorption isotherm is the equilibrium relationship between the amount of adsorbed (q_e_) and the residual adsorbate concentration (C_e_) at constant temperature^34^. In general, adsorption isotherms provide information on the affinity and the binding energy between the adsorbate and the adsorbent, on the adsorption capacity, and on the surface phase, which may be considered as a monolayer or multilayer^35^. The modelling of the adsorption isotherms consists of describing the exponential data using theoretical or empirical mathematical equations and allowing the determination of isotherm parameters to compare the efficiency of different adsorbents. The adsorption isotherm investigates the relationship between the amount of dye adsorbed onto an adsorbent and the concentration of dye in the liquid phase^8^.

The empirical Freundlich model, which is good for low concentrations and is based on sorption on the uneven surface, is shown by the following equation:

8 $${q}_{e}={K}_{F}{{C}_{e}}^{1/n},$$

where Ce (mg/L) is the equilibrium concentration of adsorbate, qe (mg/g) is the amount of adsorption at equilibrium. K_F_ and n are Freundlich constants related to the adsorption capacity and adsorption intensity, respectively. The Freundlich model is an empirical equation based on the solute distribution at equilibrium between the solid and aqueous phases^36^. The Freundlich model is applied to heterogeneous surfaces, but it can only describe adsorption data over a limited range^9^.

All the parameters of the isotherm models were calculated from nonlinear fitting of q_e_ versus C_e_ in Origin Lab. The Temkin model reflects the properties of indirect adsorbate-adsorbent interactions on the adsorption isotherm. It assumes that the heat of adsorption of all molecules in the layer decreases linearly with coverage due to adsorbate-adsorbent interactions^36^. Furthermore, a uniform distribution of binding energies, up to a maximum binding energy, characterises adsorption^6^. The Temkin model is expressed by the following equation:

9 $${q}_{e}=\frac{RT}{b}\mathrm{ln}\left(A{C}_{e}\right),$$

q_e_ (mg/g) represents the amount of adsorption at equilibrium, C_e_ (mg/L) represents the equilibrium concentration of adsorbate, T (K) represents the temperature in Kelvin, R (J/mol/K) represents the universal gas constant, b (J/mol) represents the heat of adsorption, and K_T_ represents the equilibrium binding constant proportional to the maximum binding energy.

**Figure 10:**
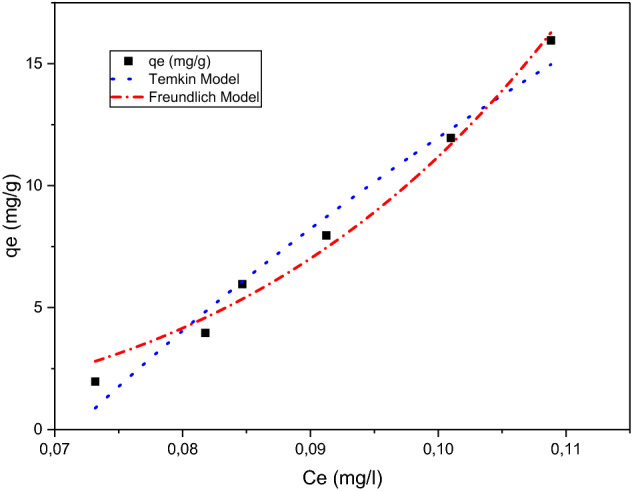
Fitting of adsorption isotherms into experimental data.

Figure 10 illustrates the curves of Freundlich and Temkin using Eqs. 7 and 8, respectively. Table 4 depicts calculated parameters of Freundlich and Temkin isotherms, R^2^ values, obtained by the nonlinear fitting method. Based on the R^2^ value comparisons, the Freundlich model represents a better fit of experimental data at equilibrium compared to the Temkin model. Thus, the adsorption process of methylene blue can be best described by the Freundlich isotherm, which indicates the multilayer adsorption on the heterogenous surface with a different energy distribution^7^. The Freundlich constant, n, is a measure of adsorption intensity^1^. A value of 1/n was found to be between 0 and 1, indicating the favourable adsorption of methylene blue^37^.

**Table 4:** Temkin and Freundlich isotherm parameters.

Temkin Model			Freundlich		
b	K_T_	R^2^	1/n	K_F_	R^2^
69.85	14.01	0.97	0.23	303192.56	0.99

### Adsorption mechanism

The adsorption rate was governed a multistep elementary reaction mechanism, since intraparticle diffusion was not the only rate-limiting step of the adsorption process of methylene blue onto iron supported by carbon foam but that other mechanisms may also control the rate of the adsorption, all of which may be operating simultaneously^38^. Also, as the Freundlich isotherm was the best fit for this study and is used to represent adsorption processes that occur on heterogeneous surfaces and active sites with varying energies based on multilayer adsorption and equilibrium, it was the most appropriate model for this investigation. These complicated processes may involve lateral contacts, numerous binding sites, and/or non-random adsorbate contributions. In recent years, it has been considered that multi-step adsorption contributes to the estimation of the adsorption rate.

### Flow-through apparatus

The performance of the flow-through apparatus was studied by examining breakthrough curves and saturation points of the sorbent. Breakthrough curves represent the ratio of effluent concentration to initial concentration over a specified period. A flow-through apparatus acts as a fixed bed column where the bed is made up of an adsorbent. The treated water flows through the fixed bed and the contaminants adhere to the surface of the adsorbent, releasing the treated water into the environment. The detoxication and recycling processes present in the textile industry must be implemented on a large scale due to the exorbitant amount of water required for finishing processes. Therefore, the efficiency and cost-effectiveness of treatment processes become a major consideration. The breakthrough point is defined as the phenomenon when the solute begins to appear in the effluent, while the exhaustion point occurs when the outflow concentration reaches the inflow concentration^1^. The studies were carried out at room temperature.

The transport of solute from bulk solution through liquid film to the adsorbent exterior surface is represented by the first portion of the plot on the left. The second part is attributed to intraparticle diffusion as a slower process. The slope of the linear part shows the rate of the adsorption process; the lower slope corresponds to a slower adsorption process as expressed by^39^. Thus, initially at the rate of diffusion of methylene blue molecules, they are quickly adsorbed onto the interior of adsorbent particles. Last, the last part is due to the final equilibrium stage, when the intraparticle diffusion slows down because there isn’t much dye in the water.

### Effect of flowrate

**Figure 11:**
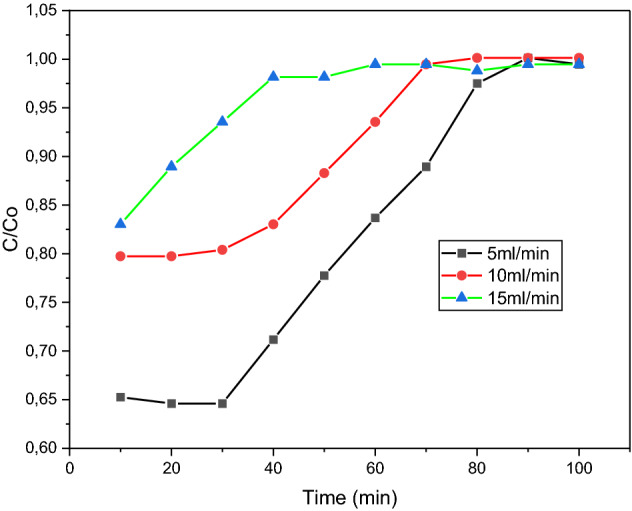
Flowrate breakthrough curve for 6 wt% iron.

Variation of the flowrate was achieved by altering the speed of the peristaltic pump. For this test, three-speed settings of 5 mL/min, 10 mL/min, and 15 mL/min were used for this test, whilst the concentration and bed height were kept constant at 10 mg/L and 2 cm, respectively. Figure 11 provides a visual representation of the time taken for the concentration of the effluent to begin to increase to its original concentration. This point in time is referred to as the “breakthrough time”. The trend portrayed by Fig. 11 suggests that an increase in flow rate resulted in a decrease in the breakthrough time. At 5 mL/min, the breakthrough time was between 30 and 40 min, whereas, for the 15 mL/min run, the breakthrough occurred much sooner, between 10 to 20 min. A consequence of higher flowrates is the decrease in contact time between the dye molecules and the adsorbent. This significantly reduces the amount of adsorption occurring as the solution passes through a fixed bed. This results in an increased effluent concentration, which can be seen by the decreasing gradient of the curves in Fig. 11 as the flowrate increases. The faster breakthrough can also be explained in terms of mass transfer, where it is known that mass transfer increases with increasing flowrates and this results in poor residence time. This then leads to increased effluent concentration and faster breakthrough^40^.

**Figure 12:**
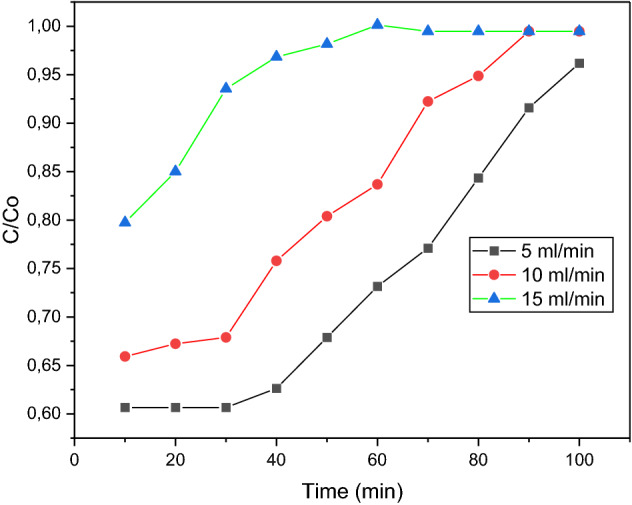
Flowrate breakthrough curve for 15 wt% iron.

The sample of 15 wt% iron shown in Fig. 12, all 3 flowrates, showed additional dye removal compared to the sample of 6 wt% iron in Fig. 11. This agrees with the conclusion of the iron content test, where it was reported that sorbents of higher metal loadings displayed increased adsorption capabilities. The gradients in Fig. 11 appear to level out at the point of saturation for each of the tested flowrates. This did not occur in Fig. 12 for the 5 mL/min flowrate, thus indicating that saturation was not reached within 100 min. Although the dye removal was superior for the sample of 15 wt% iron, there was little to no improvement in the breakthrough points or each flowrate as they occurred within the same time ranges as the sample of 5 wt% iron. Even though the breakthrough points didn’t change for the sample with 15 wt% iron, sample B was better at adsorption because it removed more dye and could be used for longer.

### Effect of bed height

A breakthrough curve can be obtained when using a flow-through apparatus. This curve illustrates the relationship between time and the concentration of the adsorbate (contaminant) in the effluent stream. Initially, when the solution begins to flow through the column, rapid adsorption should occur as the driving force for adsorption is largest at this point due to the absence of contaminants on the sorbent surface. This should result in a low concentration of adsorbate in the effluent. The breakthrough point refers to the point at which the concentration of the adsorbate in the effluent begins to rise. This is an indication of a decrease in the driving force for adsorption. Once the concentration of the effluent approaches the concentration of the original solution, the sorbent is said to be approaching its saturation point. When saturation is near, the concentration of the effluent is the same as the concentration of the original solution. At this point, no more adsorptions can happen.

The bed heights used for this test were 1 cm, 2 cm, and 3 cm, whilst other variables such as methylene blue dye concentration and flowrate were kept constant at 10 mg/L and 10 mL/min, respectively. At 10-min intervals, cuvettes were used to collect the effluent from the tube, and these samples were tested with the spectrophotometer. Three readings were taken, and average values were calculated. This procedure was repeated for bed heights of 2 cm and 3 cm.

**Figure 13:**
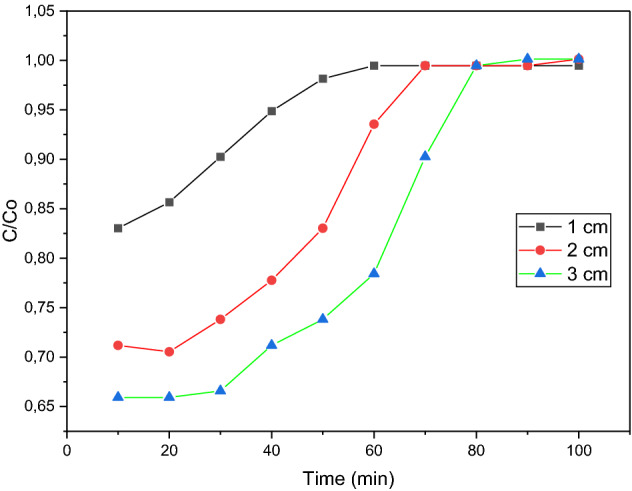
Height breakthrough curve for 6 wt% iron.

Figure 13 illustrates the relationship between time and the amount of organic dye in the effluent for each of the 3-bed heights investigated. It was observed that an increase in bed height resulted in an increase in the breakthrough time. This is plausible because at greater bed heights, the solution remains in contact with the sorbent for a longer duration, which results in a greater percentage of dye removal. Higher bed heights imply more sorbent. Hence, as with the sorbent dosage test, the increase in surface area increases the availability of adsorption sites, thereby increasing the adsorption efficiency. The slope of the breakthrough curve was found to increase with increasing bed height. In terms of mass transfer, this can be attributed to the increased length of the mass transfer zone. The breakthrough time increased from the range of 10–20 min to 30–40 min as the bed height increased from 1 to 3 cm. Therefore, to obtain an effluent of lower concentration for a longer time, a larger bed height was necessary. Because there is more adsorbent in a bed with a higher height, the breakthrough and equilibrium adsorption rates go up.

**Figure 14:**
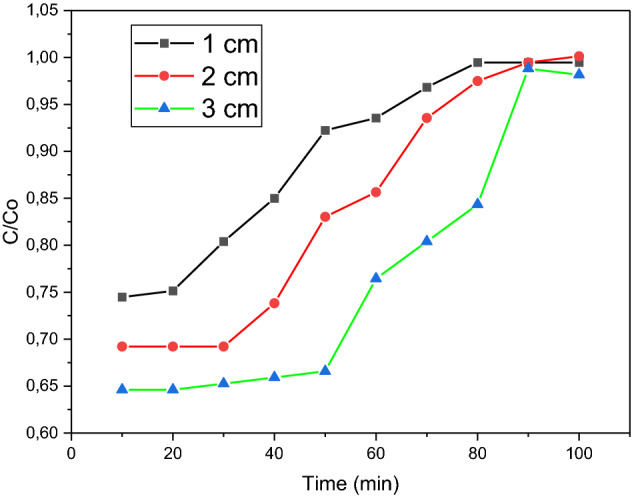
Height breakthrough curve for 15 wt% iron.

The increased iron content of the sample at 15 wt% increased the amount of adsorption occurring at bed heights of 1 cm and 2 cm. It was observed that the breakthrough time for these bed heights also increased. Figure 14 shows that the breakthrough occurred between 20 and 30 min for the 1 cm bed, whereas for sample A, at the same bed height, the breakthrough occurred between 10 and 20 min. This is a result of the increased iron content, which increases the surface area available for adsorption. For the 3 cm run, it was observed that initially the same amount of dye was adsorbed as sample A. This was implied by the C/Co ratio, which reflected a value of 0.65 on Figs. 13, 14. As the flowrate was constant at 10 mL/min for both runs, this may have impacted the adsorption of dye molecules by limiting the interaction between the molecules and the sorbent. Therefore, the limited contact time may have inhibited the dye adsorption for the 15 wt% iron sample. Figure 14 showed that the breakthrough occurred between 50 and 60 min for a 15 wt% iron sample, which was much later than the breakthrough for a sample of 6 wt% iron, which was between 30 and 40 min. Refer to Fig. 11. It should also be noted that the saturation points of the 15 wt% sorbent were reached much later than the 6 wt% sample. The 3 cm bed height failed to reach saturation in the 100-min testing period. Therefore, the 15 wt. Due to the higher metal loading of the sorbent, the 1% sample was better at attracting metal than the 6 wt% sample.

### Industrial application of flow-through apparatus

Since the addition of chemical compounds is not a requirement in the separation process, adsorption in fixed bed columns is one of the most widely used industrial processes for the removal of contaminants from aqueous textile effluents^41^. The sorbent bed generally comprises activated carbon as this is the most effective sorbent for the treatment of textile effluents due to its large surface area. On an industrial scale, the treatment of wastewater through adsorption can be problematic as most sorbents are selected based on adsorption potential, which is directly related to the size of the sorbent particles. The best performing sorbents are generally those that have the greatest surface area^42^. To have a large surface area, the size of the sorbent particles would have to be minuscule. This then presents the issue of sorbent recovery. In large bodies of water, minute particles will be difficult to retrieve. The use of support will aid in recovery as multiple sorbent particles will accumulate on the support, making it easier to retrieve. However, the use of a flow-through apparatus will alleviate the need for sorbent recovery. The sorbent will be confined to the column and, once the water has been treated, it can be released directly into the environment. The spent sorbent can be renewed by simply replacing the fixed bed.

Recovery of sorbents after they have been released into wastewater becomes problematic when using traditional batch adsorption techniques, which refer to the treatment of a fixed volume of water using a fixed amount of sorbent^43^. Therefore, to alleviate the need for sorbent recovery, flow through adsorption systems is being investigated. The advantage of using the flow-through apparatus is that this technique of wastewater treatment is relatively inexpensive and simple to implement. In the fixed bed system used for this experiment, the sorbent was confined to the column and the dye solution continuously flowed through the sorbent bed at a constant flowrate. This process of dye removal is viable for large-scale industrial use because the large quantities of industrial wastewater would be difficult to treat using conventional batch sorption methods. Flow-through systems make sure that the dye molecules are always in contact with a certain amount of new adsorbent. This makes sure that the dye is taken out as well as possible^43^.

### Thermodynamic analysis

Thermodynamic parameters such as the Gibbs energy (∆G), enthalpy (∆H), and entropy (∆S) are the actual indicators for the adsorption process’s practical application. Which process will occur spontaneously can be predicted based on the values of these parameters^3^. The following equations^3^ were used to calculate the thermodynamic parameters:

10 $$\Delta G= \Delta H-T\Delta S,$$

11 $${K}_{c }= \frac{{q}_{e}}{{C}_{e}},$$

12 $$\mathrm{ln}{K}_{c} =\frac{\Delta S}{R}- \frac{\Delta H}{RT},$$

13 $$\Delta G= -RT\mathrm{ln}{K}_{c},$$

where K_c_, R, and T represent the equilibrium constant, the amount of dye adsorbed on the adsorbent of the solution at equilibrium (mol/L), the equilibrium concentration of dye in the solution (mol/L), the gas constant (8.314 J/mol K), and the absolute temperature (K). The slopes and intercepts of a graph of ln Kc versus 1/T can be used to estimate the ∆H and ∆S values. The Gibbs free energy of change is used to evaluate the spontaneity and feasibility of adsorption processes. Both 15 wt% and 6 wt% iron samples yielded a positive ∆G^0^ value, 6.671 kJ/mol and 3.943 kJ/mol, which is indicative of a non-spontaneous process.

## Conclusion

Iron oxide immobilised on naturally derived carbon foam exhibits reasonable adsorption capabilities for the removal of organic dyes from aqueous solutions. Adsorption studies revealed that increasing the iron dosage resulted in the highest removal efficiency. Adsorption isotherm and kinetic model studies revealed that the Langmuir isotherm and pseudo-first order kinetic models provided the best fit for the study. The percentage of dye removal for the 15 wt% iron content was 9.21% greater than that of the 6 wt% sample, which indicated that sorbent efficiency increased with increasing iron content. Contact time and sorbent dosage tests showed greater adsorption occurring at higher concentrations of dye solution as a result of greater concentration gradients. Amplifying sorbent dosage enhanced the adsorptive properties of the sorbent due to the increased availability of adsorption sites. Increased contact time allowed for sorbent saturation to occur, therefore maximising the adsorption potential of the sorbent. Increased bed height imposes a longer contact time between the dye and sorbent, therefore increasing breakthrough time. Higher flowrates reduced the efficiency of the sorbent and decreased the breakthrough time as contact time between dye molecules and the surface of the sorbent was reduced. The use of the flow-through apparatus is inexpensive and alleviates the need for sorbent recovery. It is suitable for large quantities of water, which makes it suitable for large-scale industrial use. The data showed that the adsorption process could be improved by using lower flowrates and increasing the contact time, concentration of the dye solution, iron content of the sample, and sorbent dosage. Transmission electron microscopy (TEM) and Brunauer–Emmett–Teller (BET) techniques were utilised to evaluate the physical characteristics and surface morphology of synthesised carbon foam. The adsorption rate was governed by a multistep elementary reaction mechanism, as intraparticle diffusion was not the only rate-limiting step in the adsorption process of methylene blue onto iron supported by carbon foam, but that other mechanisms may also control the adsorption rate, with all of them operating simultaneously. This investigation was best represented by the pseudo-first order kinetic model (R^2^ > 0.96), while the Freundlich isotherm best describes the adsorption equilibrium (R^2^ > 0.99). Results indicated that an iron oxide sorbent immobilised on natural carbon foam is an effective sorbent for removing methylene dye.

## Data Availability

All information used to support the outcomes of this study are included within the article.
